# Study on the Mechanism of Buyang Huanwu Decoction in Treating Ischemic Stroke by Regulating the NLRP3/Caspase-1 Signaling Pathway

**DOI:** 10.3390/ph19040567

**Published:** 2026-04-01

**Authors:** Keqi Zeng, Cong Nie, Xin Zhou, Die Pei, Jieyi Huang, Yingfeng Zhang

**Affiliations:** School of Chinese Materia Medica, Guangzhou University of Chinese Medicine, University Town, Panyu District, Guangzhou 510006, China

**Keywords:** Buyang Huanwu Decoction, ischemic stroke, NLRP3/Caspase-1 pathway, OGD/R, cerebrospinal fluid

## Abstract

**Aim:** This study investigates how Buyang Huanwu Decoction (BHD) protects against cerebral ischemic damage by targeting the NLRP3/Caspase-1 pathway. **Methods:** The fingerprint of BHD was analyzed by HPLC-UV. Migratory chemicals in BHD-containing cerebrospinal fluid (BHD-CCSF) were analyzed by ultra-performance liquid chromatography-quadrupole-time of flight-mass spectrometry (UPLC-Q-TOF-MS). The effects of BHD on the NLRP3/Caspase-1 pathway, IL-18 and IL-1β levels in oxygen and glucose deprivation/reperfusion (OGD/R) cells were assessed by Western blot and ELISA. Cerebral infarction severity in permanent middle cerebral artery occlusion (pMCAO) mice was assessed by mNSS scores and staining. Protein and mRNA levels of the NLRP3/Caspase-1 pathway and inflammatory factors (IL-18, IL-1β) were measured. **Results:** BHD-containing serum (BHD-CS), BHD-CCSF, and Calycosin (Cal) reduced NLRP3, Caspase-1, ASC, GSDMD proteins, IL-18 and IL-1β in OGD/R cells. In pMCAO mice, BHD decreased pathway-related proteins and mRNA and inflammatory factors and alleviated brain injury. **Conclusions:** BHD ameliorates cerebral ischemia by inhibiting the NLRP3/Caspase-1 pathway, thereby suppressing pyroptosis and inflammation.

## 1. Introduction

Ischemic stroke (IS) is a life-threatening cerebrovascular condition with an elevated prevalence of disability and fatality [1]. More than 70% of patients suffer from neurological dysfunctions [2]. The prevailing clinical approach to treating stroke involves thrombolysis or mechanical embolectomy to reestablish blood vessel patency and reinstate cerebral blood flow [3]. Research indicates a close association between inflammation and neurological disorders [4,5], with inflammatory responses playing a significant role in the pathological progression of IS. Inflammatory reactions lead to disruption of the blood–brain barrier (BBB) and upregulation of cell adhesion molecules, enabling immune cells such as neutrophils to infiltrate brain tissue. This further activates inflammatory cascades, ultimately resulting in neuronal death [6,7]. Pyroptosis constitutes a pivotal inflammatory injury mechanism within the pathological progression of IS. Pyroptosis is categorized into classical and non-classical pyroptotic pathways based on their dependent factors and mediating pathways. Notably, cytoplasmic engulfment of inflammasomes is induced by the classical pyroptotic pathway represented by NLRP3/Caspase-1 [8]. Following IS, NLRP3 exposes its effector domain and forms a complex with ASC and pro-caspase-1. This complex cleaves the dimeric pro-caspase-1 into two subunits, p10 and p20, generating active Caspase-1. This activates IL-1β and IL-18, which further induce the release of inflammatory mediators, triggering a cascade-like inflammatory response. On the other hand, GSDMD, a key effector molecule in pyroptosis, releases its active N-terminus after its C-terminus is recognized and bound with high affinity by activated caspase-1 p20/p10. This active N-terminus then targets and binds to lipid membranes, polymerizing into a hollow ring-shaped oligomer that forms a non-selective pore. This leads to cellular swelling and ultimately triggers pyroptosis [9,10,11]. Inhibition of NLRP3 inflammation-related pro-death can significantly improve the neurological function of the cerebral ischemic injury model and reduce the infarct size and edema degree [12].

Buyang Huanwu Decoction (BHD), a classic prescription known for promoting blood circulation and replenishing Qi, is widely used to treat cerebral ischemic injury. BHD can be used to treat IS by reducing inflammation and inhibiting excitatory amino acid toxicity, neuronal apoptosis and anti-oxidative stress [13,14,15]. Calycosin (Cal), the primary active constituent of *Astragali Radix*, exhibits estrogen-like properties and demonstrates a specific neuroprotective effect against cerebral ischemic injury, thereby reducing neuronal damage [16,17].

Different from the single structure of chemical drugs, the selection of test subjects of BHD with multiple components coexisting is very important. Single components are not sufficient to fully reflect the characteristics of therapeutic efficacy of BHD. In view of the specificity of the stroke lesions’ location and the action of BHD, this research simultaneously uses BHD-containing serum (BHD-CS), BHD-cerebrospinal fluid (BHD-CCSF), and Cal for cell experiments.

In this study, following the preparation and implementation of quality control measures for the lyophilized product of BHD, the migration components of cerebrospinal fluid (CSF) in rabbits were identified. Combined with the oxygen and glucose deprivation/reperfusion (OGD/R) cell and permanent middle cerebral artery occlusion (pMCAO) mouse models, we studied the action mechanism of the anti-cerebral ischemia of BHD-CS, BHD-CCSF, and Cal based on the NLRP3/Caspase-1 pathway.

Signaling pathways are an effective and widely used research method for the mechanism of action of traditional Chinese medicine (TCM). The pathogenesis of stroke is very complex, and BHD as a complex system exerts therapeutic effects involving numerous signaling pathways. Literature reports indicate that the primary signaling pathways through which BHD intervenes in IS include the Cav-1/Notch1/Hes1 pathway, the PI3K/Akt pathway, and the TLR4/MyD88/NF-κB inflammatory pathway. Inflammation plays a central role in cerebral ischemic injury after stroke, and the NLRP3/Caspase-1 pathway is a classic inflammatory pathway and is completely different from the abovementioned signaling pathway. The NLRP3/Caspase-1 signaling pathway holds a special position in the inflammatory response of stroke. However, the mechanism of BHD in intervening in the NLRP3/Caspase-1 signaling pathway against IS has not been deeply studied. Therefore, combine cellular and animal experiments to conduct an in-depth study on the protective mechanism of BHD in intervening in the NLRP3/Caspase-1 signaling pathway against cerebral ischemic injury to enrich the protective mechanism of BHD against IS, aiming to provide reference for its rational clinical application.

## 2. Results

### 2.1. Fingerprint Analysis of Lyophilized Product of BHD

The fingerprint results are shown in Figure S1. Cal was determined as the reference peak for this fingerprint. Compared with the reference substance, Hydroxysafflor Yellow A, Albiflorin, Paeoniflorin, Ononin, Ferulic acid, Calycosin-7-O-glucoside, Calycosin, Benzoylpaeoniflorin, Formononetin, and Senkyunolide are identified in the lyophilized product of BHD. The results showed that the similarity between the 16 batches of BHD is more than 0.97. The cluster analysis results are shown in Figure S2.

### 2.2. Analysis of BHD Lyophilized Product CSF-Migration Components

**Figure 1:**
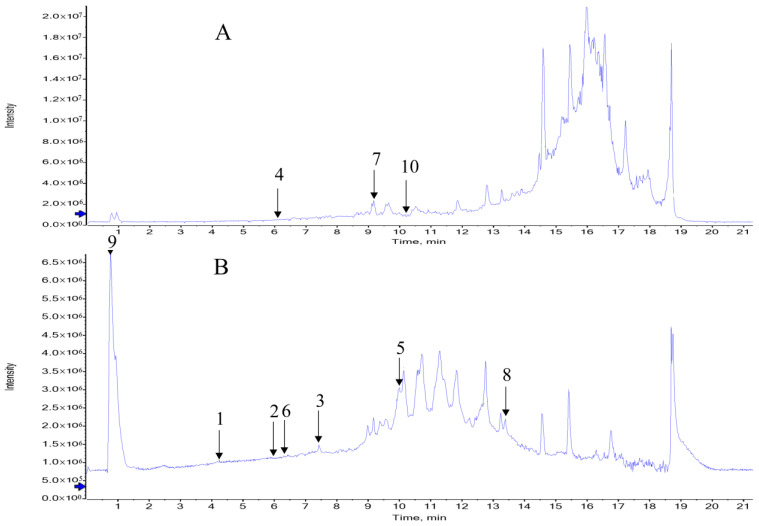
Total ion chromatogram of rabbit drug-containing CSF. The numbers represent the corresponding compounds identified in the CSF of rabbits in Table 1. (**A**) Positive ion mode; (**B**) negative ion mode.

The total ion current chromatogram of CSF in rabbits is shown in Figure 1, and the rabbit CSF-migrating compounds of BHD are shown in Table 1.

### 2.3. BHD Protected OGD/R HT22 Cell Injury

**Figure 2:**
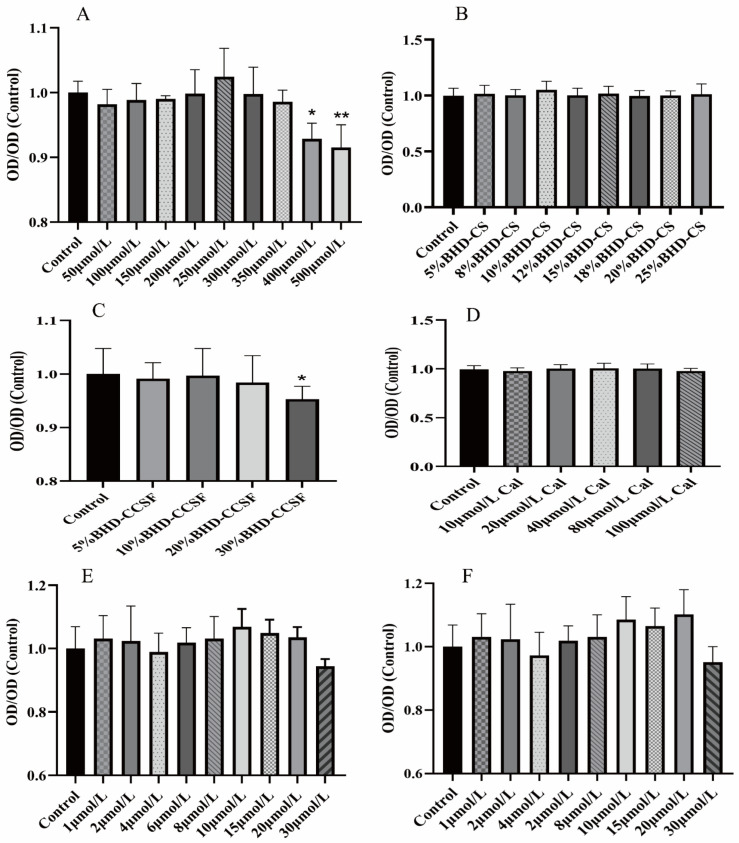
CCK-8 assay for the survival rate of normal HT22 cells. (**A**) EDA; (**B**) BHD-CS; (**C**) BHD-CCSF; (**D**) Cal; (**E**) MCC950; (**F**) DSF. The data are presented as mean ± SD (*n* = 6). * *p* < 0.05, ** *p* < 0.01 compared with the control group.

The cell viability of HT22 cells was 56% after 8 h of hypoxia and 24 h of reoxygenation, which was the best condition for the OGD/R modeling of HT22 cells. The non-toxic dose ranges of EDA, BHD-CS, BHD-CCSF, and Cal on normal HT22 cells were determined, and the results are shown in Figure 2. BHD-CS concentrations were determined as 2.5%, 5%, and 10%, representing low, medium, and high levels, respectively. BHD-CCSF was present in concentrations of 2.5%, 5%, and 10%, categorized as low, middle, and high, respectively. Cal concentrations ranged from 20 μmol/L (low) to 80 μmol/L (high), with a middle concentration of 40 μmol/L. The optimal dose of MCC950 was determined to be 10 μmol/L, the administration time was 24 h, and the optimal dose of Disulfiram (DSF) was 15 μmol/L.

**Figure 3:**
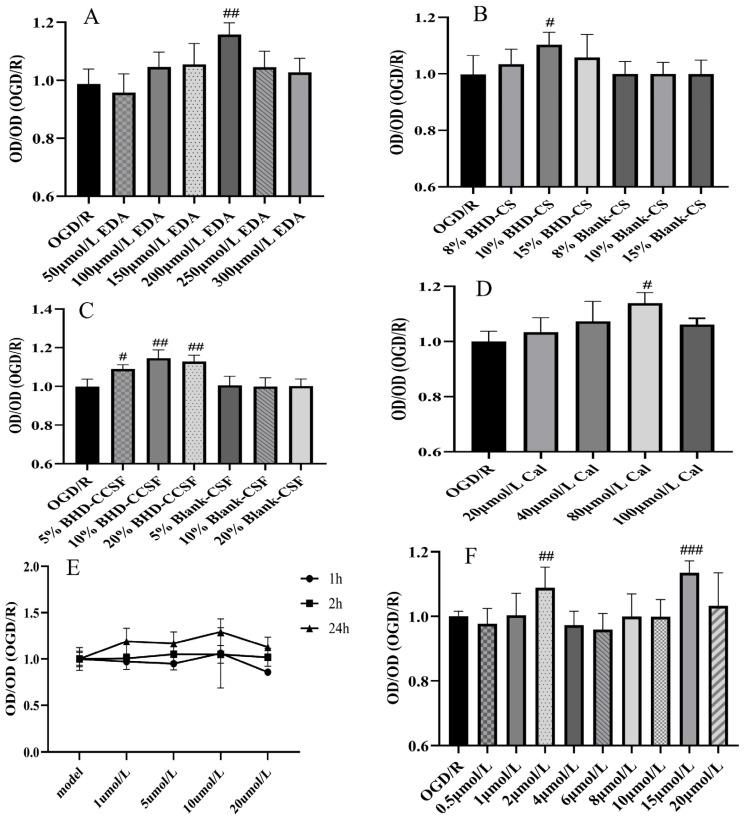
Effects of EDA, BHD-CS, BHD-CCSF, Cal, MCC950, and DSF on OGD/R HT22 cell viability. (**A**) EDA; (**B**) BHD-CS; (**C**) BHD-CCSF; (**D**) Cal; (**E**) MCC950; (**F**) DSF. The data are presented as mean ± SD (*n* = 6). ^#^ *p* < 0.05, ^##^ *p* < 0.01 and ^###^ *p* < 0.001 compared with the OGD/R group.

Cell viability in the OGD/R group decreased significantly compared with the control group (*p* < 0.001). In contrast, treatment with BHD-CS, BHD-CCSF, EDA, and Cal significantly increased cell viability in a dose-dependent manner (*p* < 0.05). Furthermore, the BHD-CS and BHD-CCSF groups displayed the most pronounced effect on cell survival rate. These aforementioned results are exhibited in Figure 3.

**Figure 4:**
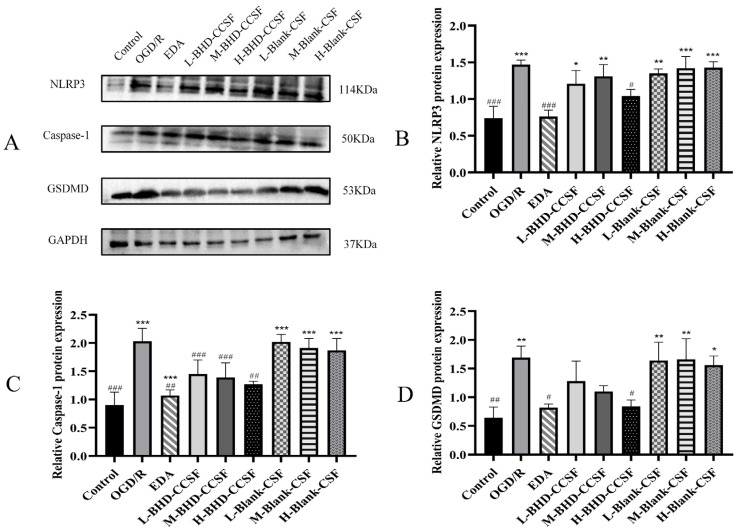
The impact of BHD-CCSF on OGD/R HT22 cells. (**A**) Representative WB bands; (**B**) NLRP3; (**C**) Caspase-1; (**D**) GSDMD. GAPDH was utilized as the reference protein, and normalize the target protein to GAPDH. The data are presented as mean ± SD (*n* = 6). * *p* < 0.05, ** *p* < 0.01 and *** *p* < 0.001 compared with the control group. ^#^ *p* < 0.05, ^##^ *p* < 0.01 and ^###^ *p* < 0.001 compared with the OGD/R group.

**Figure 5:**
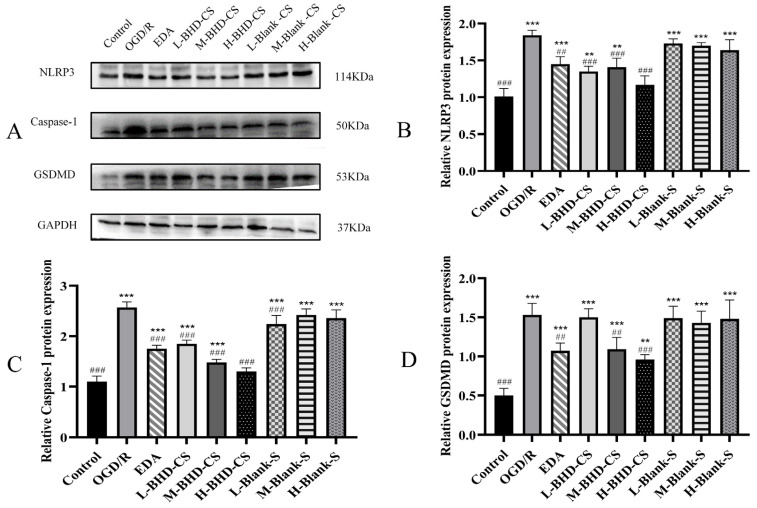
The impact of BHD-CS on OGD/R HT22 cells. (**A**) Representative WB bands; (**B**) NLRP3; (**C**) Caspase-1; (**D**) GSDMD. GAPDH was utilized as the reference protein, and normalize the target protein to GAPDH. The data are presented as mean ± SD (*n* = 6). ** *p* < 0.01 and *** *p* < 0.001 compared with the control group. ^##^ *p* < 0.01 and ^###^ *p* < 0.001 compared with the OGD/R group.

**Figure 6:**
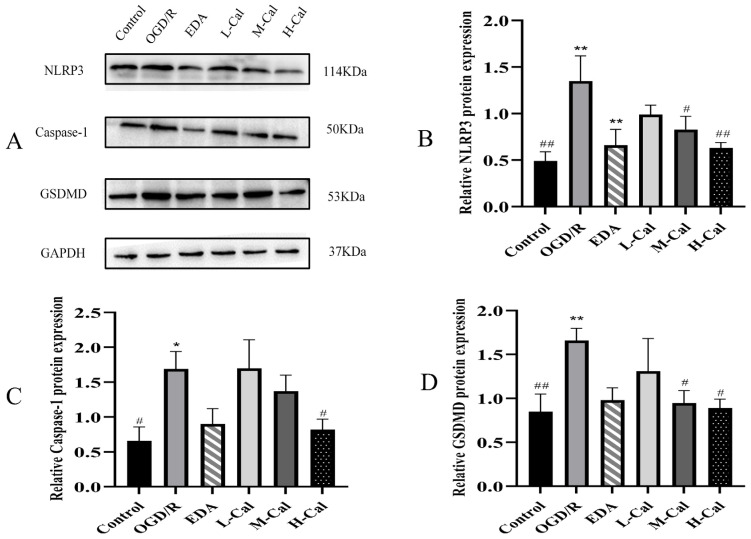
The impact of Cal on OGD/R HT22 cells. (**A**) Representative WB bands; (**B**) NLRP3; (**C**) Caspase-1; (**D**) GSDMD. GAPDH was utilized as the reference protein, and normalize the target protein to GAPDH. The data are presented as mean ± SD (*n* = 6). * *p* < 0.05 and ** *p* < 0.01 compared with the control group. ^#^ *p* < 0.05 and ^##^ *p* < 0.01 compared with the OGD/R group.

Expression levels of NLRP3, Caspase-1, and GSDMD were significantly upregulated in the model group compared with the control group (*p* < 0.05). Moreover, the levels of GSDMD, Caspase-1, and NLRP3 decreased as the concentrations of EDA, BHD-CS, BHD-CCSF, and Cal increased. The aforementioned results are displayed in Figure 4, Figure 5 and Figure 6.

### 2.4. Effect of BHD-CS and BHD-CCSF on NLRP3, Caspase-1, GSDMD, Caspase-1 p20 and ASC Expression Levels in OGD/R HT22 Cell

**Figure 7:**
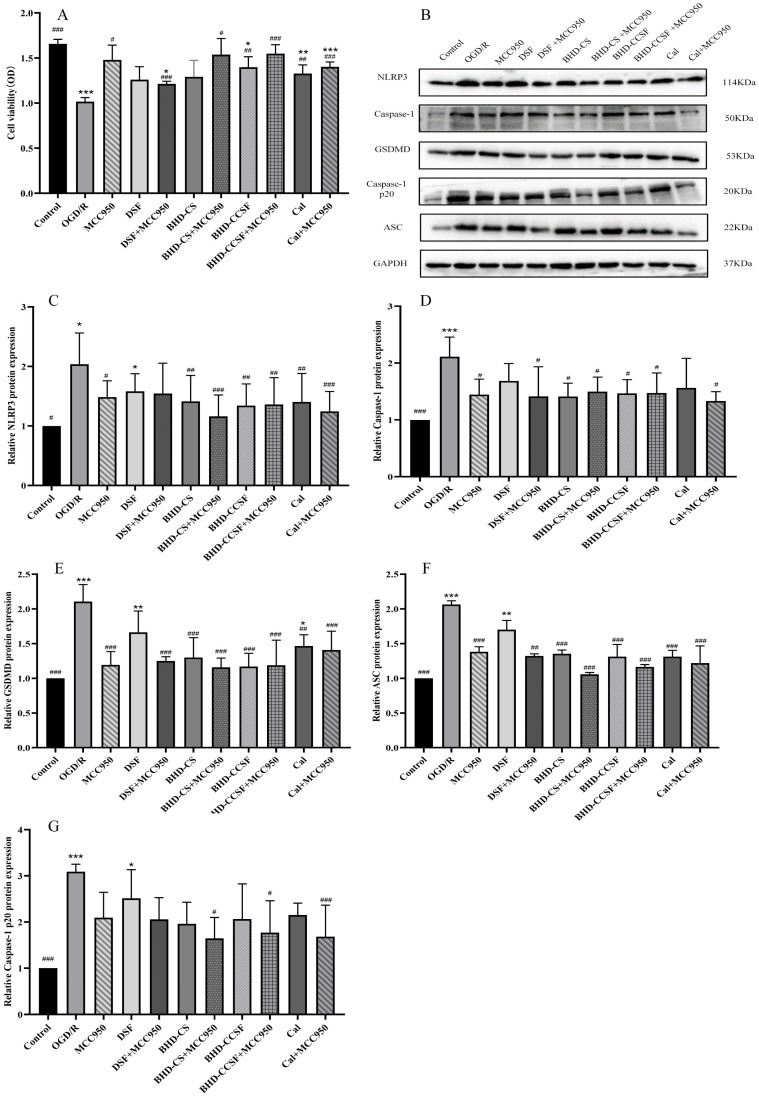
Mechanism study on the intervention of BHD in the NLRP3/Caspase-1 signaling pathway. (**A**) OGD/R HT22 cell viability; (**B**) representative WB bands; (**C**) NLRP3; (**D**) Caspase-1; (**E**) GSDMD; (**F**) ASC; (**G**) Caspase-1 p20. GAPDH was utilized as the reference protein, and normalize the target protein to GAPDH. The data are presented as mean ± SD (*n* = 6). * *p* < 0.05, ** *p* < 0.01 and *** *p* < 0.001 compared with the control group. ^#^ *p* < 0.05, ^##^ *p* < 0.01 and ^###^ *p* < 0.001 compared with the OGD/R group.

The expression levels of GSDMD, Caspase-1, NLRP3, Caspase-1 p20, and ASC proteins were notably up-regulated in the ten additional groups versus the control group (*p* < 0.05). Additionally, the BHD-CCSF, BHD-CS, and Cal groups demonstrate a noticeable decrease in the expression of the protein mentioned above when juxtaposed with the OGD/R group. Furthermore, the combination with MCC950 further reduced the protein expression levels in each group. The data gathered is shown in Figure 7.

### 2.5. Effect of BHD on the Level of IL-1β and IL-18 in the OGD/R HT22 Cell

**Figure 8:**
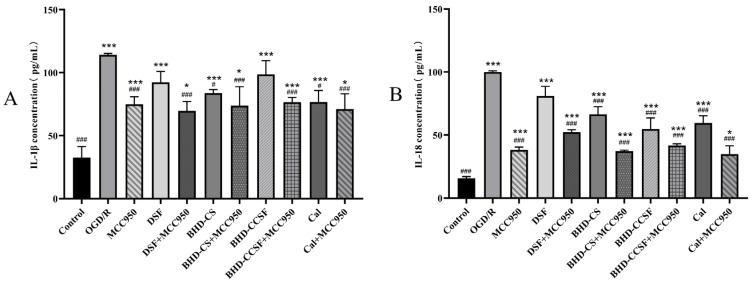
The expressions of IL-1β and IL-18 in OGD/R HT22 cells. (**A**) IL-1β; (**B**) IL-18. The data are presented as mean ± SD (*n* = 6). * *p* < 0.05 and *** *p* < 0.001 compared with the control group. ^#^ *p* < 0.05 and ^###^ *p* < 0.001 compared with the OGD/R group.

All groups exhibited a noticeable increase in the expressions of IL-18 and IL-1β (*p* < 0.05) when compared with the control group. However, a notable reduction in the expression levels of IL-1β and IL-18 was recorded in all groups, except the control group, in juxtaposition with the OGD/R group (*p* < 0.05). The results are depicted in Figure 8.

### 2.6. Neuroprotective Effect of BHD on the pMCAO Mice

**Figure 9:**
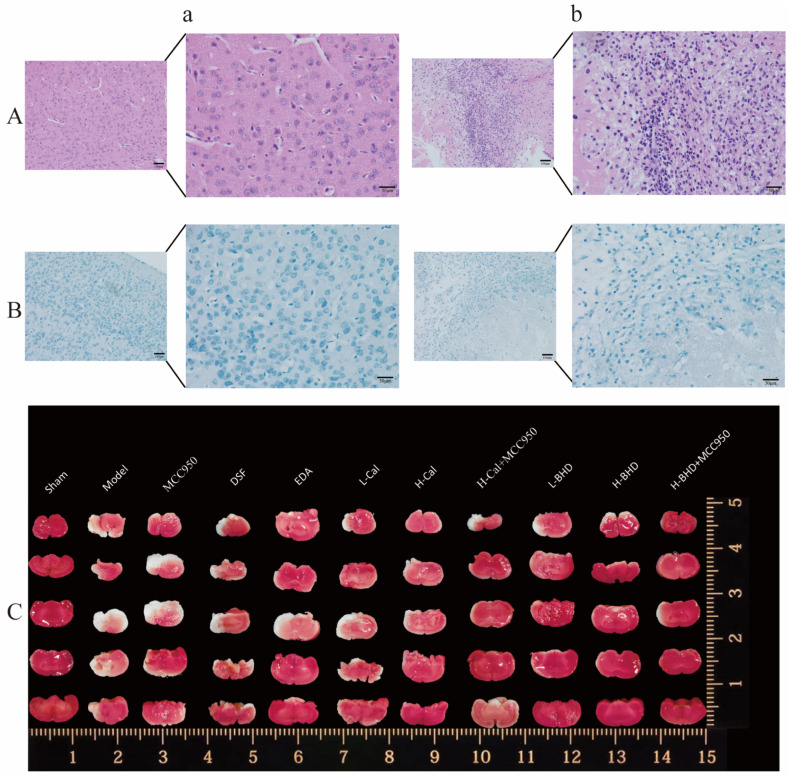
Staining of mouse brain. (**A**) Nissl staining; (**B**) HE staining; (**C**) TTC staining; (**a**) control group; (**b**) model group.The overall and partially enlarged image scales are 50 and 100 microns, respectively.

HE staining results (Figure 9A) and Nissl staining (Figure 9B) showed that nerve cells were evenly distributed in the sham group, with normal neuron structure and clear hierarchy, while in the model group, the distribution of nerve cells was disordered, the cells were swollen and ruptured, the cell boundaries of most neurons were unclear, and the nucleus was atrophied. Combining the results of the two dyeing methods indicated that the pMCAO model was established successfully. As shown in Figure 9C, the brain non-infarcted areas are bright red, while the infarcted areas are pale white.

**Figure 10:**
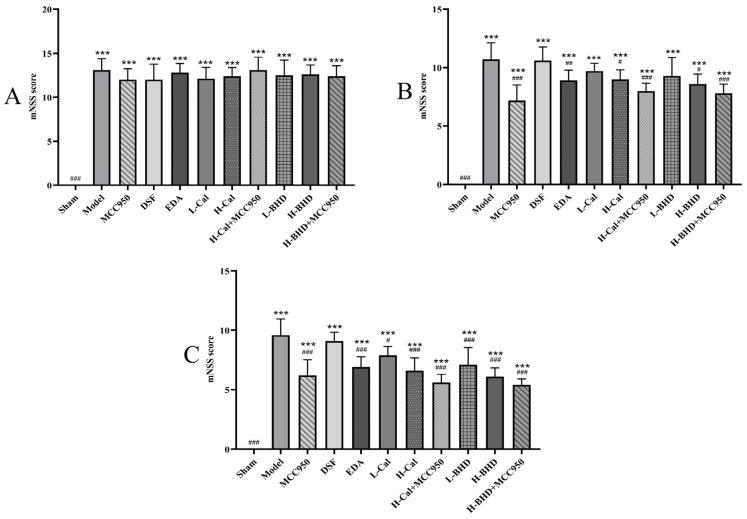
The mNSS score of mice. (**A**) day 1; (**B**) day 7; (**C**) day 14. The data are presented as mean ± SD (*n* = 10). *** *p* < 0.001 compared with the sham group. ^#^ *p* < 0.05, ^##^ *p* < 0.01 and ^###^ *p* < 0.001 compared with the model group.

The mNSS scores in the sham group demonstrated an extensive decrease compared to the other groups on days 1, 7, and 14 after the pMCAO modeling (*p* < 0.05). Specifically, in contrast to the results for the model group, the seventh-day scores of the MCC950 group, high-dose Cal group, high-dose Cal+MCC950 group, high-dose BHD group, and high-dose BHD+ MCC950 group all showed a marked decrease (*p* < 0.05). Similarly, the scores of all groups, except the DSF and the sham group, demonstrated a notable decrease in comparison to the model group on the 14th day (*p* < 0.05). The results are displayed in Figure 10.

### 2.7. Effects of BHD on the Expressions of NLRP3, Caspase-1, GSDMD, Caspase-1 p20, and ASC in pMCAO Mice

**Figure 11:**
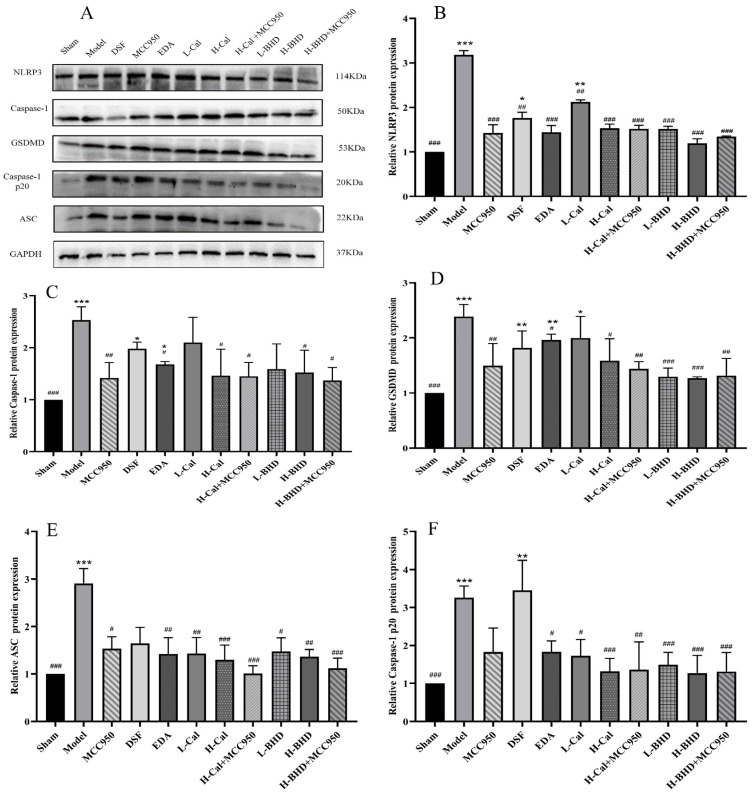
Relative expression of proteins in mouse brain tissue. (**A**) WB bands; (**B**) NLRP3; (**C**) Caspase-1; (**D**) GSDMD; (**E**) ASC; (**F**) Caspase-1 p20. GAPDH was used as the reference protein, and normalize the target protein to GAPDH. The data are presented as mean ± SD (*n* = 6). * *p* < 0.05, ** *p* < 0.01 and *** *p* < 0.001 compared with the control group. ^#^ *p* < 0.05, ^##^ *p* < 0.01 and ^###^ *p* < 0.001 compared with the OGD/R group.

The expression of all the proteins mentioned above was notably increased in the model group compared to the sham group (*p* < 0.001). Among them, the expressions of GSDMD and Caspase-1 in the model group were approximately 2.5 times that of the sham group, while the expression levels of NLRP3, Caspase-1 p20 and ASC in the model group were approximately 3 to 3.2 times that of the sham operation group. However, when compared to the model group, the expression levels of GSDMD, NLRP3, Caspase-1, Caspase-1 p20, and ASC in all groups were significantly lower (*p* < 0.05). Compared with the model group, after administration of Cal, BHD, and EDA, the expression levels of GSDMD and Caspase-1 were all reduced by approximately 20–40%, while the expression levels of NLRP3, Caspase-1 p20 and ASC in the model group were all reduced by approximately 40–60%. The results are shown in Figure 11.

### 2.8. Effect of BHD on the Level of IL-18 and IL-1β in the Ischemic Penumbra of pMCAO Mice

**Figure 12:**
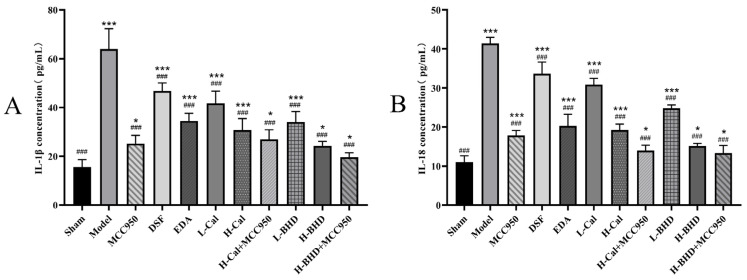
Expression levels of inflammatory cytokines in pMCAO mouse brain tissue. (**A**) IL-1β; (**B**) IL-18. The data are presented as mean ± SD (*n* = 6). * *p* < 0.05 and *** *p* < 0.001 compared with the control group. ^###^ *p* < 0.001 compared with the OGD/R group.

The expression levels of IL-18 and IL-1β were considerably higher in the other groups compared to the sham group. The expression levels of the aforementioned inflammatory factors in the model group were approximately 4 times that of the sham group. In comparison with the model group, all groups exhibited significant reductions in the expression of inflammatory factors mentioned above (*p* < 0.05). Compared with the model group, after administration of Cal, BHD, and EDA, the expression levels of the above inflammatory factors was reduced by approximately 30–60%. The results are illustrated in Figure 12.

### 2.9. Effect of BHD on NLRP3 and GSDMD mRNA Expression

**Figure 13:**
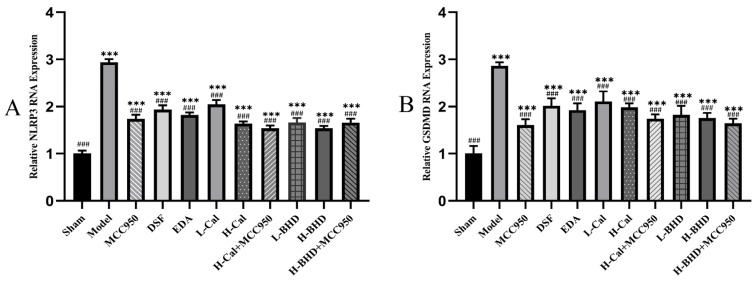
NLRP3 and GSDMD mRNA expression levels in pMCAO mouse brain tissue. (**A**) NLRP3; (**B**) GSDMD. The data are presented as mean ± SD (*n* = 6). *** *p* < 0.001 compared with the control group. ^###^ *p* < 0.001 compared with the OGD/R group.

The PCR amplification curves of NLRP3, GSDMD, and the internal reference GAPDH mRNA displayed an “s” shape, with the Ct values falling within the range of 12–30, indicating normal amplification. The melting curves were unimodal, with a Tm range of 80–90 °C, suggesting the high specificity of the designed primers and the absence of non-specific amplification. Furthermore, compared to the sham group, the other groups’ GSDMD and NLRP3 mRNA expression levels were notably higher. The expression levels of the aforementioned mRNA in the model group were approximately 3 times that of the sham group, while the expression levels in the other groups were approximately twice that of the sham group. In comparison to the model group, all groups exhibited a significant reduction in mRNA expressions of NLRP3 and GSDMD. Except for the sham group, the expression levels of NLRP3 and GSDMD mRNA in the other groups were approximately 60% of those in the model group. The results are shown in Figure 13.

## 3. Discussion

BHD originates from the Qing dynasty medical text “Medical Forest Correcting Errors” and is regarded within TCM as a classic prescription for stroke-related conditions [18]. Modern research indicates that BHD is widely employed in IS. BHD exerts neuroprotective effects against IS by inhibiting inflammation, reducing oxidative stress, and suppressing apoptosis. Concurrently, it demonstrates therapeutic potential through enhancing neuroplasticity, promoting angiogenesis, and mitigating excitotoxicity [19]. Ci Song et al. [20] established rat models of pMCAO and OGD/R in rat brain microvascular endothelial cells, which demonstrated that BHD suppresses IS injury by inhibiting glycolysis and apoptosis through downregulating pan-kla and H3K18la levels and Apaf-1 transcriptional activity.

As a compound decoction of TCM, BHD’s therapeutic effect on IS stems from the combined action of multiple components and targets. Consequently, this study investigated the holistic pharmacological effect rather than the efficacy of any single constituent [21,22]. At the pharmacological level, TCM’s distinctive feature lies in multi-component, multi-target systemic regulation. Single herbs or compound formulas typically involve multiple active constituents acting synergistically, exerting therapeutic effects by modulating multiple signaling pathway networks. This represents a marked contrast to the single-component, single-target mode of action.

As BHD is traditionally administered as a decoction, which poses storage challenges, it was prepared as a lyophilized powder for animal experiments. This study employed HPLC-UV technology to establish a fingerprint spectrum for the lyophilized powder, enabling comprehensive quality control of the powder. In the preliminary stage, we employed ultra-performance liquid chromatography-quadrupole-time of flight-mass spectrometry (UPLC-Q-TOF-MS) technology to identify the components in BHD lyophilized powder and those that migrated into the blood and brain of rats [23]. Therefore, we further identified the components of BHD-CCSF in this paper, laying the groundwork for the subsequent application of BHD-CCSF in cellular therapy and establishing the material basis for BHD’s treatment of IS.

The chemical makeup of TCM is intricate, and there is biological transformation that occurs after the drug is introduced into the body, which leads to a distinct difference between the components of TCM that migrate in the blood and brain and those found in the original herbal medicine [24]. Active ingredients need to be distributed to various tissues and organs via the bloodstream to play a therapeutic role, and BHD-CS can reflect the pharmacological effects of drugs to a certain extent; therefore, the introduction of serum pharmacochemistry has enhanced the research methods and approaches in the field of TCM. Our previous research found that after intragastric administration of BHD in pMCAO rats, 44 compounds entered the blood and 24 compounds entered the brain. Furthermore, eight components were identified in the rat CSF [23]. The medicinal components identified in BHD-CCSF are considered potential active constituents. Yaru Pan et al. [25] replicated the MCAO model, demonstrating that Ferulic acid and Amygdalin could be detected in rat brains via LC-MS analysis. Research [26,27,28,29] has revealed that Cal, an isoflavone in the BHD, constitutes a key active component for treating IS. Consequently, building upon the BHD study, we further selected Cal and BHD-CCSF for investigation.

CSF is a colorless, transparent fluid found in the subarachnoid space, ventricles, and central canal of the spinal cord and is essential for nourishing brain cells and maintaining intracranial pressure. It exchanges substances closely with the brain tissue and is the best research object for the study of central nervous system diseases, and its composition can reflect the transport and action of drugs in the brain [30]. The central nervous system possesses unique physiological and anatomical structures. The closed nature of the brain and the existence of the BBB restrict the transfer of blood-borne components. For neurological diseases such as IS, where both the lesion site and drug action site are located in the brain, the BBB, constructed by microvascular endothelial cells and astrocytes, significantly differentiates the microenvironment where neurons survive from serum. BHD-CCSF can more accurately reflect the situation where drugs, after absorption and metabolism, act on the central nervous system through the BBB. BHD-CCSF holds certain advantages in pharmacological studies of the nervous system. It can simulate the process of BHD components penetrating the BBB and entering the central nervous system, addressing the inherent limitation of medicated serum, which overlooks the screening effect of the BBB. This allows *in vitro* experimental results to more accurately reflect the efficacy of TCM in the brain. CSF pharmacology has become a powerful tool in stroke research. Tongqiao Huoxue Decoction-containing CSF has been found to inhibit apoptosis of OGD/R HT22 cells through the ASK1/MKK4/JNK pathway, providing a protective effect against I/R injury [31]. Additionally, the Dandeng Tongnao-containing CSF has been observed to have a protective effect on OGD/R BMECs in rats [32]. Currently, the safeguarding impact of BHD-CCSF on cerebral ischemic injury has been rarely reported. Therefore, we conducted *in vitro* experiments using BHD-CCSF and BHD-CS to examine their potential anti-cerebral ischemia properties. Our results suggest that BHD-CCSF and BHD-CS may improve OGD/R-injured HT22 cells.

BHD demonstrates a protective effect on the nervous system, with inflammation playing a vital role in pathological advancement [33,34]. Our results show that both BHD and Cal can effectively improve the survival rate of OGD/R-injured HT22 cells, reduce the neural function score, and improve neural function. The protective benefits of BHD and Cal against brain ischemia damage have been investigated further through the anti-inflammatory effects on the CNS both *in vivo* and *in vitro*. We have demonstrated that BHD may inhibit the NLRP3/Caspase-1 signaling pathway and has a neuroprotective effect on IS.

Inflammation is a crucial defense mechanism for the body to maintain homeostasis and respond to injury, but its dysregulation serves as an important pathological basis for various diseases, including stroke. IL-1β and IL-18 are the core hubs of the inflammatory signaling network, playing a crucial role in maintaining immune balance and mediating pathological inflammation. IL-1β and IL-18 exist in the inactive forms of pro-IL-1β and pro-IL18, and their maturation relies on the activation of Caspase-1 mediated by the NLRP3 inflammasome. The inflammasome consists of NLRP3, ASC, and Caspase-1 and can sense stimuli such as LPS and viruses, promoting the maturation and release of IL-1β and IL-18. IL-1β and IL-18 are core pro-inflammatory effector molecules involved in cell pyroptosis. They are activated and released by the inflammasome-Caspase-1 pathway during pyroptosis, serving as key executors of the “inflammatory death” characteristic of pyroptosis. They must rely on being cleaved into their mature form by Caspase-1 to exert biological effects. GSDMD leads to cell membrane perforation and the release of strongly pro-inflammatory IL-1β and IL-18, which are important characteristics that distinguish pyroptosis from apoptosis and necrosis. NLRP3 activation is triggered by a variety of stimuli, including K^+^ efflux, Na^+^ inflow, Cl^−^ depletion, intracellular calcium overload, lysosome damage, and protein kinase R activation [35]. Once activated, the NLRP3 inflammasome further amplifies the inflammatory response of the nervous system. Neuron pyroptosis has been extensively linked to the pathogenic mechanisms of CNS ailments, which comprise traumatic brain injury and cerebral I/R injury [36,37]. By suppressing the NF-κB/NLRP3 axis, a PRMT5 inhibitor could mitigate pyroptosis and inflammation triggered by cerebral I/R [38]. Tongxinluo has been shown to reduce the expression levels of GSDMD, NLRP3, IL-6, and IL-1β, thereby protecting ischemic brain tissue by inhibiting both classical and non-classical pathways of pyroptosis [39]. Additionally, hydrogen sulfide has the potential to safeguard CNS against I/R-induced neuroinflammation through impeding the NLRP3/Caspase-1/GSDMD classical pyroptosis pathway [40]. The glycosides present in BHD have the potential to exert neuroprotective effects by modulating the traditional pyroptosis pathway [41]. Both BHD and Cal effectively suppress the levels of Caspase-1 and NLRP3 proteins, as well as Pro-IL-18 and IL-1β, both *in vitro* and *in vivo*, leading to an improvement in neurological function. Furthermore, when combined with the NLRP3 inhibitor MCC950, the expression levels of the pro-inflammatory cytokines and aforementioned proteins are further reduced, resulting in a more pronounced protective effect. Consequently, it can be inferred that the inhibition of the NLRP3/Caspase-1 pathway may be responsible for BHD’s anti-cerebral ischemia impact.

The selection and use of positive control drugs in pharmacological research is crucial for ensuring the quality of the study. In the research on the pharmacodynamics of TCM compounds, a single chemical drug is commonly used as the positive control, but there are also related studies using chemical drugs and proprietary Chinese medicines for double positive control. The therapy of IS has made considerable use of EDA. According to recent investigations, DSF can effectively decrease the expression of the NLRP3 inflammasome and Caspase-1, thereby achieving anti-pyroptosis and anti-inflammatory impacts [42]. Thus, drug that have been publicly listed and exhibit a clear pyroptosis inhibitory effect, DSF is used as a positive control for inhibiting the NLRP3/Caspase-1 classical pyroptosis pathway. Therefore, our study selected EDA and DSF as double positive controls for efficacy and mechanism, and the efficacy and mechanism of drug action on diseases were studied from the aspects of neuroprotection and inhibiting pyroptosis. It has a certain reference value for the selection of positive drugs in the pharmacodynamics study of Chinese compound medicine.

Numerous studies have demonstrated that Cal, the primary active component found in *Astragali Radix* in BHD, possesses anti-inflammatory, antiviral, and free radical scavenging properties, among others. As a natural flavonoid with estrogen-like effects, Cal has demonstrated good anti-cerebral ischemia injury effects in both in vitro and in vivo experiments. It has been substantiated that the combined administration of Cal and paeoniflorin can effectively mitigate brain tissue injury by modifying the PI3K/AKT signaling pathway [43]. Furthermore, our previous investigation has revealed that Cal exhibits a potential therapeutic effect in alleviating cerebral ischemic injury. In this research, it was observed that both BHD and Cal have the ability to modulate the levels of mRNA, proteins, and pro-inflammatory factors related to the NLRP3/Caspase-1 signaling pathway, thereby ameliorating the symptoms of cerebral ischemic injury.

These outcomes imply that BHD may exert a neuroprotective impact in IS by suppressing the NLRP3/Caspase-1 signaling pathway, which may help further clarify the underlying process of BHD’s anti-cerebral ischemia and offer potential insights for its clinical application.

The primary components of *Astragali Radix* include Astragaloside I, II, III, IV, and Calycosin-7-glucoside, etc. Hydroxycrocin Yellow A, Crocin, Neocrocin, Kaempferol, and other active ingredients are abundant in *Carthami Flos*. Albiflorin, Paeoniflorin, Benzoylpaeoniflorin and others were found in *Paeoniae Radix Rubra*. Senkyunolide A, 3-butylidenephthalide, Ferulic acid, and Chlorogenic acid were isolated and identified in *Angelicae Sinensis Radix*. The active components, such as Tetramethylpyrazine, Senkyunolide A, Senkyunolide I, are present in *Chuanxiong Rhizoma*. Arachidonic acid is the main component of *Pheretima*. The principal components of *Persicae Semen* include Amygdalin and Chlorogenic acid.

BHD employs a substantial dosage of *Astragali Radix*. The active component, Astragaloside IV, significantly alleviates neurological deficits in rats subjected to cerebral ischemia–reperfusion injury, as well as NLRP3 inflammasome-induced neuronal apoptosis [44]. Cal improves neurological dysfunction in rats subjected to cerebral ischemia–reperfusion injury, with therapeutic efficacy increasing with higher doses [45]. Cal enhances cardiac function and mitigates myocardial injury and fibrosis by inhibiting NLRP3 inflammasome activation and reducing IL-18 and IL-1β levels [46]. Ferulic acid and Amygdalin can prevent hemorrhagic transformation after delayed t-PA infusion via inhibiting NLRP3 inflammasome/pyroptosis associated with microglial PGC-1α [25].

BHD contains abundant glycosides and flavonoids, which may be related to the inhibition of the NLRP3 inflammatory pathway. Especially Cal, a characteristic bioactive component of *Astragali Radix* that enters the bloodstream and brain, may be closely related to the mechanism of BHD’s anti-cerebral ischemia effects by inhibiting the NLRP3/Caspase-1 signaling pathway.

## 4. Materials and Methods

### 4.1. Quality Control of BHD Lyophilized Product

#### 4.1.1. Preparation of BHD Lyophilized Product

The complete prescription of herbs and other detailed information are shown in Table 2. All herbs have been identified as genuine herbs. According to our group’s previously established methods [47], 16 batches of BHD lyophilized products were prepared.

#### 4.1.2. The Fingerprint Study of BHD Lyophilized Product Based on HPLC-UV

A total of 0.2 g of lyophilized powder was combined with 1 mL of methanol and sonicated for 20 min. After centrifuging at 10,000 rpm under 4 °C for 10 min, the supernatant was obtained. A gradient elution method employing 0.1% formic acid water (A) and acetonitrile (B) with an injection volume of 10 μL and a flow rate of 0.8 mL/min at 250 nm was used to carry out the chromatographic separation on a Phenomenex Luna C18 column (250 × 4.60 mm, 5 μm). The elution gradient procedure is shown in Table S1.

### 4.2. Rabbit BHD-CCSF and Rat BHD-CS Sampling and Migratory Component Identification

#### 4.2.1. Sampling of Rabbit BHD-CCSF and Rat BHD-CS

Sixteen male New Zealand rabbits (2 ± 0.2 kg, 6 months old) were purchased from Suibei Experimental Animal Farm, Baiyun District, Guangzhou (Animal production license: SCXK (YUE) 2020-0050). A total of twelve SD rats (330 ± 20 g, 8 weeks of age) were acquired from Zhuhai BesTest Bio-Tech Co., Ltd. (Zhuhai, China) (Animal Production License: SCXK (YUE) 2020-0051). Both rabbits and rats were divided into two groups randomly.

The animals involved in this study were reared in conditions with a regulated temperature of 25 °C, a humidity range of 30–60% and a light/dark cycle lasting for 12 h each day. All animals have clear species and genetic background. Sample sizes for all animals were guided by the 3Rs principle and ARRIVE guidelines to accomplish the purpose of the experiment with a minimum sample size.

After an acclimatization period, rabbits of the BHD group received a gavage feeding of BHD lyophilized product equivalent to 8.1 g/kg of herbal medicine for 14 days, while rats of the BHD group received BHD lyophilized product equivalent to 16.68 g/kg of herbal medicine for 7 days. The control group received saline of equal volume. An hour after the last dose, BHD-CCSF samples were collected from the rabbits, and serum was collected from the rats. To obtain the BHD-CS, the serum was inactivated at 56 °C for 30 min, using a 0.22 μm sterile filter.

#### 4.2.2. Identification of Rabbit BHD-CCSF Migration Components with UPLC-Q-TOF/MS

In total, 300 μL of BHD-CCSF was lyophilized at −80 °C; then 100 μL acetonitrile was added and sonicated for one minute at 4 °C. The mixture was centrifuged for a total of ten minutes at 10,000 rpm at 4 °C to collect the supernatant.

The component identification of BHD-CCSF was conducted using AB/SCIEX 5600+ UPLC-Q-TOF-MS (PerkinElmer Inc., Waltham, MA, USA) with an Agilent ZORBAX SB-Aq C18 column (2.1 mm × 100 mm, 3.5 μm). Table S2 illustrates the elution gradient procedure and other detailed information. In positive ion mode, the injection volume was set at 10 μL, whereas in negative ion mode, it was 20 μL. Analyst TF 1.7 software was used for data acquisition.

The TCMSP database and related literature were used to summarize the BHD component information. Peakview (2.0.0.7849) was employed to analyze the mass spectrum information of BHD and BHD-CCSF, and the endogenous components in blank CSF were deducted. The component structure was comprehensively confirmed using secondary mass spectrometry, ChmicalBook, PubChem, and TCMSP database with the error < 5 ppm between the measured and theoretical value as the identification criteria.

### 4.3. Study on the Action Mechanism of BHD-CS and BHD-CCSF in OGD/R HT22 Cell

#### 4.3.1. Construction of the OGD/R HT22 Cell Model

The cells were cultured in an anoxic environment with a 1:4:95 ratio of oxygen, nitrogen, and carbon dioxide at 37 °C for 8 h after the cell media was removed and swapped with glucose-free DMEM. For an extra day, the cells were reoxygenated in a thermostatic incubator after the high-glucose DMEM was added to substitute the glucose-free DMEM.

#### 4.3.2. CCK-8 Assay of Cell Viability

CCK-8 was used to determine cell viability in each group, and the cell-free pore was set as the blank group.

#### 4.3.3. Screening of Effective Concentration of BHD-CCSF, BHD-CS, Edaravone (EDA), Cal, MCC950 and DSF in OGD/R Cells

The HT22 cells were randomly allocated to the control group, EDA group, BHD-CS, BHD-CCSF group, Cal group, MCC950 group and DSF group. Each group was added with different concentrations of EDA (50, 100, 150, 200, 250, 300, 350, 400, 450, and 500 μmol/L, CSN pharm Corporation, CSN10476, Arlington Heights, IL, USA), BHD-CS (5%, 8%, 10%, 12%, 15%, 18%, 20%, and 25%), BHD-CCSF (5%, 10%, 20%, and 30%), Cal (10, 20, 40, 80, and 100 μmol/L, Jiangsu Yongjian Pharmaceutical Technology Co., LTD, 103403, Taizhou, China), MCC950 (1, 2, 4, 6, 8, 10, 15, 20, and 30 μmol/L, Sigma-Aldrich Corporation, PZ0280, St. Louis, MO, USA) and DSF (1, 2, 4, 6, 8, 10, 15, 20, and 30 μmol/L, Meilunbio Corporation, A0322A, Dalian, China). The effects of EDA, BHD-CS, BHD-CCSF, Cal, MCC950 and DSF on the proliferation inhibition rate of HT22 cells were assessed using the CCK-8 kit to determine the non-toxic dose range. Subsequently, the CCK-8 technique was employed to assess the influence of BHD-CS, BHD-CCSF, Cal, MCC950, and DSF groups on the survival rate of OGD/R HT22 cells, aiming to determine suitable concentrations for each group.

#### 4.3.4. Effect of BHD-CS, BHD-CCSF, and Cal on OGD/R HT22 Cells

Proteins separated on 12% SDS-PAGE gels were transferred onto PVDF membranes. Afterwards, the membranes were treated with rabbit anti-mouse primary antibodies NLRP3 (1:1000), Caspase-1 (1:1000), GSDMD (1:1000), and GAPDH (1:10,000) for an entire night at 4 °C and were then submerged in sealed liquid. Following that, the PVDF membranes were exposed to goat anti-rabbit secondary antibodies for 2 h with a dilution ratio of 1:5000 except for GAPDH (1:10,000). Image J1.8.0 was used to analyze the protein band’s gray levels.

### 4.4. Study on the Action Mechanism of BHD-CS, BHD-CCSF, and Cal in OGD/R HT22 Cells Based on the NLRP3/Caspase-1 Signaling Pathway

#### 4.4.1. Determination of Protein Expression Levels Determination of NLRP3, GSDMD, Caspase-1, Caspase-1 p20 and ASC in OGD/R HT22 Cells by WB

The WB process was the same as in Section 4.3.4, and the dilution ratio of rabbit-derived primary antibody Caspase-1 p20 and ASC was 1:1000 and 1:500, respectively.

#### 4.4.2. Determination of IL-18 and IL-1β Levels in HT22 Cells

IL-18 and IL-1β levels were determined according to the manufacturer’s instructions for the ELISA kit (Jiangsu Enzyme Free Industrial Co., Ltd., Yancheng, China).

### 4.5. Regulation Mechanism of BHD in pMCAO Mice Involved the NLRP3/Caspase-1 Signaling Pathway

#### 4.5.1. Mouse Modeling and Grouping

In total, 200 male Kunming mice of 32 ± 3 g (8 weeks of age) were obtained from Zhuhai BesTest Bio-Tech Co., Ltd. (Animal Production License: SCXK (YUE) 2020-0051). The pMCAO mouse model was replicated following Zea Longa’s [48] suture-occluded method. Zea Longa scoring was performed after modeling, and model mice with scores of 1–3 were selected for subsequent experiments. The middle cerebral artery was not blocked, and the suture was solely placed into the carotid artery for the sham group.

The mice were randomly divided into 11 groups using an Excel table to generate a random number table, namely the sham group, pMCAO group (model), MCC950 group, low-dose BHD group, high-dose BHD group, low-dose Cal group, high-dose Cal group, high-dose BHD+MCC950 group, high-dose Cal+MCC950 group, DSF group, and EDA group. Except for the sham group, which had 10 animals, all the other groups had 19 animals for the modeling process. The baseline data, such as body weight of each group of animals, were compared using one-way analysis of variance, and *p* > 0.05 indicates that the baseline is balanced and comparable. After modeling, the mice in each group were treated with the corresponding drugs according to Table 3 for 14 days and subjected to mNSS scoring on days 1, 7, and 14 after pMCAO modeling.

#### 4.5.2. Model Verification

The establishment of the pMCAO model was verified by Nissl staining and HE staining. The brain tissue was immersed in 4% paraformaldehyde for fixation, sectionalized after paraffin embedding, and then stained. The stained sections were observed under a microscope to evaluate the tissue structure and cell morphology.

#### 4.5.3. TTC Staining

The entire brain of the mice was collected and subjected to freezing at −20 °C for 30 min. TTC staining was performed after brain slicing.

#### 4.5.4. Determination of Expression Levels of NLRP3, Caspase-1, GSDMD, Caspase-1 p20, and ASC in Mice by WB

The WB process was consistent with that in Section 4.3.4. In comparison to GAPDH, which was used as the benchmark protein, the expression of NLRP3, Caspase-1, GSDMD, Caspase-1 p20, and ASC was quantified.

#### 4.5.5. Determination of IL-18 and IL-1β Levels in the Ischemic Penumbra of pMCAO Mice

The supernatant of pMCAO mouse brain tissue was obtained using a tissue grinder, and the method for the determination of IL-18 and IL-1β in the ischemic penumbra of pMCAO mice is similar to that in Section 4.4.2.

#### 4.5.6. RT-PCR Analysis

PCR primers were purchased from Sangon Biotech Co., Ltd., (Shanghai, China) and the primer sequences are shown in Table 4. Total RNA from mouse brain tissues was extracted using the TRIzol reagent (A4A3062, Thermo, Waltham, MA, USA). The Evo M-MLV kit (A4A2758, Hunan Ecoray Biological Engineering Co., Ltd., Changsha, China) was performed for reverse transcription. The SYBR Green Pro Taq HS Premix qPCR kit was employed for qPCR. As an internal benchmark, GAPDH was used while quantifying the data using the 2^−ΔΔCt^ approach.

### 4.6. Statistical Analysis

The quantitative analysis of the experimental data in this study was accomplished using SPSS 20.0 and GraphPad Prism 9.5.0. The experimental data were presented in the form of mean ± SD. Under the premise that all group values were completely in accordance with the normal distribution, we conducted a one-way ANOVA. The homogeneity of variance was tested using the Levene method, and it was found that all groups had homogeneity of variance (*p* > 0.05). The multiple comparisons between groups were analyzed using Tukey. All statistical data were plotted as bar charts using GraphPad Prism 9.5.0 software for visualization.

## 5. Conclusions

Treatment with BHD-CS, BHD-CCSF, and Cal significantly improved the viability of OGD/R-injured cells. Additionally, BHD and Cal demonstrated a notable reduction in the mNSS scores on days 7 and 14 after modeling, leading to an improvement in neurological deficit symptoms. Conversely, the levels of GSDMD, NLRP3, Caspase-1, ASC, and Caspase-1 p20 in OGD/R HT22 cells and pMCAO mice significantly increased compared to the control group, indicating the activation of the NLRP3/Caspase-1 signaling pathway following modeling, consequently leading to an elevation in Caspase-1 p20, GSDMD, and ASC expression. EDA, BHD, Cal, and MCC950 could reduce the levels of the proteins mentioned above. The expression of IL-18 and IL-1β considerably increased in OGD/R HT22 cells and pMCAO mice when juxtaposed with the control group, while it significantly decreased after intervention with EDA, BHD, Cal, and MCC950. Furthermore, EDA, BHD, Cal, and MCC950 may have the capacity to reduce the expression of NLRP3 and GSDMD mRNA in the brain tissue of pMCAO mice. Therefore, it is plausible that EDA, BHD, and Cal could potentially exhibit similar effects to the specific NLRP3 inhibitor MCC950 by diminishing the levels of proteins associated with the NLRP3/Caspase-1 signaling pathway, as well as the levels of inflammatory factors mentioned above in OGD/R HT22 cells and pMCAO mice.

## Figures and Tables

**Table 1:** Composition of CSF in rabbits under positive and negative ionization modes of BHD.

No.	t_R_/ min	Formula	MW	MS/ (*m*/*z*)	Additional Ion	Error (ppm)	Secondary Fragment	Name	Source
1	4.68	C_10_H_10_O_4_	194.0574	193.0508	[M-H]^−^	0.6	81/193	Ferulic Acid	a/b/e/f
2	6.00	C_10_H_18_O_4_	202.1200	201.1132	[M-H]^−^	−0.2	139/201	Sebacic acid	b
3	7.36	C_11_H_14_O	162.1040	161.0974	[M-H]^−^	1.5	83/105/161	Valerophenone	e
4	6.13	C_12_H_12_O_2_	188.0832	189.0907	[M+H]^+^	−1.7	128/189	N-Butylidenephthalide	e/b
5	9.75	C_12_H_24_O_2_	200.1771	199.1677	[M-H]^−^	3.9	67/162/199	Lauric acid	c/f
6	6.36	C_16_H_12_O_5_	284.0679	283.0604	[M-H]^−^	−2.7	211/239/268/283	Calycosin	a
7	9.05	C_16_H_22_O_4_	278.1513	279.1583	[M+H]^+^	−2.8	149/230/279	Senkyunolide Q	e
8	13.37	C_18_H_36_O_2_	284.2710	283.2634	[M-H]^−^	−3.2	265/283	Stearic acid	f/e
9	0.98	C_9_H_10_O_3_	166.0625	165.0558	[M-H]^−^	0.3	122/135/150/165	Paeonol	c
10	10.15	C_29_H_50_O	414.3856	437.3738	[M+Na]^+^	−3.6	81/108/397/437	Beta-Sitosterol	f/b/e/d

a: *Astragali Radix*; b: *Angelicae Sinensis Radix*; c: *Paeoniae Radix Rubra*; d: *Persicae Semen*; e: *Chuanxiong Rhizoma*; f: *Carthami Flos*.

**Table 2:** Composition and proportion of BHD.

Materials	Chinese Name	Latin Name	Medicinal Parts	Percentage of Total Weight
*Astragali Radix*	Huangqi	*Astragalus membranaceus* (Fisch.) Bge.	Roots	83.9
*Angelicae Sinensis Radix*	Danggui	*Angelica sinensis* (Oliv.) Diels	Roots	4.2
*Paeoniae Radix Rubra*	Chishao	*Paeonia lactiflora* Pall.	Roots	3.5
*Chuanxiong Rhizoma*	Chuanxiong	*Ligusticum chuanxiong* Hort.	Rhizomas	2.1
*Persicae Semen*	Taoren	*Prunus persica* (L.) Batsch	Seeds	2.1
*Carthami Flos*	Honghua	*Carthamus tinctorius* L.	Flowers	2.1
*Pheretima*	Dilong	*Pheretima aspergillum* (E. Perrier)	Bodies	2.1

**Table 3:** Administration dosage in each group (g/kg/d).

Group	Normal Saline	MCC950	EDA	DSF	BHD	Cal
Sham	0.9%	-	-	-	-	-
Model	0.9%	-	-	-	-	-
MCC950	-	0.05	-	-	-	-
EDA	-	-	0.01	-	-	-
DSF	-	-	-	0.015	-	-
L-BHD	-	-	-	-	14.3	-
H-BHD	-	-	-	-	57.2	-
H-BHD+MCC950	-	0.05	-	-	57.2	-
L-Cal	-	-	-	-	-	0.00176
H-Cal	-	-	-	-	-	0.00704
H-Cal+MCC950	-	0.05	-	-	-	0.00704

**Table 4:** Primers used for RT-PCR.

Gene	Species	Primer Sequence	GC Ratio (%)	Fragment Size (bp)
*NLRP3*	mouse	Forward: 5′-GGCTGCTATCTGGAGGAACTT-3′	52.38%	119
		Reverse: 5′-CATCTTCAGCAGCAGCCCTT-3′	55.00%	
*GSDMD*	mouse	Forward: 5′-TTCCAGTGCCTCCATGAATGT-3′	47.62%	193
		Reverse: 5′-GCTGTGGACCTCAGTGATCT-3′	55.00%	
*GAPDH*	mouse	Forward: 5′-GGTTGTCTCCTGCGACTTCA-3′	55.00%	183
		Reverse: 5′-TGGTCCAGGGTTTCTTACTCC-3′	52.38%	

## Data Availability

Data is contained within the article.

## Supplementary Materials

The following supporting information can be downloaded at: https://www.mdpi.com/article/10.3390/ph19040567/s1, Figure S1. HPLC fingerprint of BHD; Figure S2. Systematic cluster analysis of 16 batches of BHD; Table S1. The fingerprint gradient elution procedur of BHD lyophilized product; Table S2. The gradient elution procedur of rabbit’s BHD-CCSF detection.

## Abbreviations

BHD	Buyang Huanwu Decoction
BHD-CS	BHD-containing serum
Cal	Calycosin
CCA	Common carotid artery
DMSO	Dimethyl sulfoxide
DSF	Disulfiram
ELISA	Enzyme linked immunosorbent assay
EDA	Edaravone
BBB	blood–brain barrier
TCM	Traditional Chinese Medicine
UPLC-Q-TOF-MS	Ultra-performance liquid chromatography-quadrupole-time of flight-mass spectrometry
GSDMD	Gasdermin D
I/R	Ischemia–reperfusion
mNSS	Modified neurological severity score
OGD/R	oxygen and glucose deprivation/reperfusion
pMCAO	Permanent middle cerebral artery occlusion
BHD-CCSF	BHD-containing cerebrospinal fluid
TTC	2,3,5-triphenyltetrazolium chloride
CSF	cerebrospinal fluid
IS	ischemic stroke

## References

[1] Zhao Y., Zhang X., Chen X., Wei Y. (2022). Neuronal Injuries in Cerebral Infarction and Ischemic Stroke: From Mechanisms to Treatment (Review). Int. J. Mol. Med..

[2] Virani S.S., Alonso A., Benjamin E.J., Bittencourt M.S., Callaway C.W., Carson A.P., Chamberlain A.M., Chang A.R., Cheng S., Delling F.N. (2020). Heart Disease and Stroke Statistics—2020 Update: A Report From the American Heart Association. Circulation.

[3] Jadhav A.P., Mokin M., Ortega-Gutierrez S., Diogo H., Liebeskind D., Nogueira R., Jovin T., Linfante I. (2020). An Appraisal of the 2018 Guidelines for the Early Management of Patients with Acute Ischemic Stroke. Interv. Neurol..

[4] Kwon H.S., Koh S.-H. (2020). Neuroinflammation in Neurodegenerative Disorders: The Roles of Microglia and Astrocytes. Transl. Neurodegener..

[5] Rajesh Y., Kanneganti T.-D. (2022). Innate Immune Cell Death in Neuroinflammation and Alzheimer’s Disease. Cells.

[6] Alsbrook D.L., Di Napoli M., Bhatia K., Biller J., Andalib S., Hinduja A., Rodrigues R., Rodriguez M., Sabbagh S.Y., Selim M. (2023). Neuroinflammation in Acute Ischemic and Hemorrhagic Stroke. Curr. Neurol. Neurosci. Rep..

[7] Zhao B., Yin Q., Li Y. (2021). Research Progress of Mechanisms and Drug Therapy for Inflammation in Ischemic Stroke. Prog. Pharm. Sci..

[8] Shen J., Zhu Y., Huang K., Jiang H., Shi C., Xiong X., Zhan R., Pan J. (2016). Buyang Huanwu Decoction Attenuates H_2_O_2_-Induced Apoptosis by Inhibiting Reactive Oxygen Species-Mediated Mitochondrial Dysfunction Pathway in Human Umbilical Vein Endothelial Cells. BMC Complement. Altern. Med..

[9] Shi J., Gao W., Shao F. (2017). Pyroptosis: Gasdermin-Mediated Programmed Necrotic Cell Death. Trends Biochem. Sci..

[10] Han Y.-H., Liu X.-D., Jin M.-H., Sun H.-N., Kwon T. (2023). Role of NLRP3 Inflammasome-Mediated Neuronal Pyroptosis and Neuroinflammation in Neurodegenerative Diseases. Inflamm. Res..

[11] Lü Y. (2020). The NLRP-3/Caspase-1/GSDMD Signalling Axis Mediates Pyroptosis in Microglia. Master’s Thesis.

[12] An P., Xie J., Qiu S., Liu Y., Wang J., Xiu X., Li L., Tang M. (2019). Hispidulin Exhibits Neuroprotective Activities against Cerebral Ischemia Reperfusion Injury through Suppressing NLRP3-Mediated Pyroptosis. Life Sci..

[13] Jin Y.L., Dong L.Y., Wu C.Q., Qin J., Li S., Wang C.Y., Shao X., Huang D.K. (2013). Buyang Huanwu Decoction Fraction Protects against Cerebral Ischemia/Reperfusion Injury by Attenuating the Inflammatory Response and Cellular Apoptosis. Neural Regen. Res..

[14] Zhao Y., Ma X., Yu W., Zhang Z., Wang W., Zhou X., Gao W. (2021). Protective Effect of Buyang Huanwu Decoction on Cerebral Ischemia Reperfusion Injury by Alleviating Autophagy in the Ischemic Penumbra. Evid.-Based Complement. Altern. Med..

[15] Yin M., Liu Z., Wang J., Gao W. (2023). Buyang Huanwu Decoction Alleviates Oxidative Injury of Cerebral Ischemia-Reperfusion through PKCε/Nrf2 Signaling Pathway. J. Ethnopharmacol..

[16] Wang Y., Ren Q., Zhang X., Lu H., Chen J. (2018). Neuroprotective Mechanisms of Calycosin Against Focal Cerebral Ischemia and Reperfusion Injury in Rats. Cell. Physiol. Biochem..

[17] Liu H., Zhao Z., Yan M., Zhang Q., Jiang T., Xue J. (2023). Calycosin Decreases Cerebral Ischemia/Reperfusion Injury by Suppressing ACSL4-Dependent Ferroptosis. Arch. Biochem. Biophys..

[18] Fu S., Tang W., Luo S., Wang W., Huang Y., Li H., Wang G. (2026). Identification of Diagnostic Biomarkers for Ischemic Stroke and Drug Targets of Buyang Huanwu Decoction: A Bioinformatics and Network Pharmacology Study. Medicine.

[19] Qin Y., Hu S., Mawen S., Pan S., Huai Y., Liang G., Chen T., Zhao F., Dong H., Yao X. (2025). Neuroprotective Mechanisms of Buyang Huanwu Decoction in Ischemic Stroke. Front. Pharmacol..

[20] Song C., Fang X., Fang N., Hu F. (2024). Buyang Huanwu Decoction Suppresses Ischemic Stroke by Suppressing Glycolysis and Cell Apoptosis in Rat Brain Microvascular Endothelial Cells. Brain Res. Bull..

[21] Zhao M., Xiao L., Chen Q., Shen L., Zhao G., Linghu K., Ma Q., Dar P., Yu H. (2025). Bioactive Equivalent Combinatorial Compounds Contributing to the Holistic Effect of Traditional Chinese Medicine Bupleuri Chinense DC. J. Ethnopharmacol..

[22] Liao W., Wang P., He Y., Liu Z., Wang L. (2024). Investigation of the Underlying Mechanism of Buyang Huanwu Decoction in Ischemic Stroke by Integrating Systems Pharmacology-Proteomics and in Vivo Experiments. Fitoterapia.

[23] Zeng K., Zhou X., Nie C., Zhang Y. (2023). A Serum Pharmacochemical Study on the Pharmacodynamic Substances Basis of the Anti-Ischemic Injury of Buyang Huanwu Decoction. New Tradit. Chin. Med. Clin. Pharmacol..

[24] Tang M., Gao X., Geng T., Chen X., Wang J., Shen C., Gao H., Qian M., Wang Z., Cao L. (2021). Metabolomics Analysis of the Therapeutic Effects of Qiwei Tongbi Oral Liquid on Rheumatoid Arthritis in Rats. J. Pharm. Biomed. Anal..

[25] Pan Y., Nie L., Chen W., Guan D., Li Y., Yang C., Duan L., Wan T., Zhuang L., Lai J. (2025). Buyang Huanwu Decoction Prevents Hemorrhagic Transformation after Delayed T-PA Infusion via Inhibiting NLRP3 Inflammasome/Pyroptosis Associated with Microglial PGC-1α. J. Ethnopharmacol..

[26] Xu S., Huang P., Yang J., Du H., Wan H., He Y. (2023). Calycosin Alleviates Cerebral Ischemia/Reperfusion Injury by Repressing Autophagy via STAT3/FOXO3a Signaling Pathway. Phytomedicine.

[27] Hsu C.-C., Kuo T.-W., Liu W.-P., Chang C.-P., Lin H.-J. (2020). Calycosin Preserves BDNF/TrkB Signaling and Reduces Post-Stroke Neurological Injury after Cerebral Ischemia by Reducing Accumulation of Hypertrophic and TNF-α-Containing Microglia in Rats. J. Neuroimmune Pharmacol..

[28] Zhou Y., Song W., Wang Y., Li S., Shan C., Dong J., Xu Z., Zou H., Pan Y., Chen X. (2025). Calycosin Regulates Gut Microbiota-Bile Acid-FXR Axis to Protect Rats from Cerebral Ischemia-Reperfusion Injury. Eur. J. Pharmacol..

[29] Yang X., Pan Y., Cai L., Wang W., Zhai X., Zhang Y., Wu Q., Chen J., Zhang C., Wang Y. (2024). Calycosin Ameliorates Neuroinflammation via TLR4-Mediated Signal Following Cerebral Ischemia/Reperfusion Injury in Vivo and in Vitro. J. Inflamm. Res..

[30] Orešković D., Radoš M., Klarica M. (2017). Role of Choroid Plexus in Cerebrospinal Fluid Hydrodynamics. Neuroscience.

[31] Yuan M., Zhang Y., Wang G., Wu Q., Wang Y., Wang N. (2022). Protective Effect of Tongqiao Huoxue Decoction Containing Cerebrospinal Fluid On OGD/R-Damaged HT22 Cells via Regulation of ASK1/MKK4/JNK Signaling Pathway. China J. Chin. Mater. Medica.

[32] Gao Z., Li D., Wang N., Hou J., Zhai Y. (2018). Protective Effect of Drug-Containing Cerebrospinal Fluid of Dandengtongnao Capsule on Cerebral Microvascular Endothelial Cells in Rats Injured by OGD/R. Chin. Tradit. Pat. Med..

[33] Pan R., Cai J., Zhan L., Guo Y., Huang R.Y., Li X., Zhou M., Xu D., Zhan J., Chen H. (2017). Buyang Huanwu Decoction Facilitates Neurorehabilitation through an Improvement of Synaptic Plasticity in Cerebral Ischemic Rats. BMC Complement. Altern. Med..

[34] Chen X., Chen H., He Y., Fu S., Liu H., Wang Q., Shen J. (2020). Proteomics-Guided Study on Buyang Huanwu Decoction for Its Neuroprotective and Neurogenic Mechanisms for Transient Ischemic Stroke: Involvements of EGFR/PI3K/Akt/Bad/14-3-3 and Jak2/Stat3/Cyclin D1 Signaling Cascades. Mol. Neurobiol..

[35] He Y., Hara H., Núñez G. (2016). Mechanism and Regulation of NLRP3 Inflammasome Activation. Trends Biochem. Sci..

[36] Mortezaee K., Khanlarkhani N., Beyer C., Zendedel A. (2018). Inflammasome: Its Role in Traumatic Brain and Spinal Cord Injury. J. Cell. Physiol..

[37] Ye A., Li W., Zhou L., Ao L., Fang W., Li Y. (2020). Targeting Pyroptosis to Regulate Ischemic Stroke Injury: Molecular Mechanisms and Preclinical Evidences. Brain Res. Bull..

[38] Wu X., Wang B., Li J., Yang Z., Zhou Y., Ma X., Kou Z., Jiang L., Song J. (2022). Inhibition of PRMT5 Attenuates Cerebral Ischemia/Reperfusion–Induced Inflammation and Pyroptosis through Suppression of NF–ΚB/NLRP3 Axis. Neurosci. Lett..

[39] Jiang X., Ma C., Gao Y., Cui H., Zheng Y., Li J.X., Zong W., Zhang Q. (2023). Tongxinluo Attenuates Atherosclerosis by Inhibiting ROS/NLRP3/Caspase-1-Mediated Endothelial Cell Pyroptosis. J. Ethnopharmacol..

[40] Yang K.L., Li W.H., Liu Y.J., Wei Y.J., Ren Y.K., Mai C.D., Zhang S.Y., Zuo Y., Sun Z.Z., Li D.L. (2022). Hydrogen Sulfide Attenuates Neuroinflammation by Inhibiting the NLRP3/Caspase-1/GSDMD Pathway in Retina or Brain Neuron Following Rat Ischemia/Reperfusion. Brain Sci..

[41] She Y., Shao L., Zhang Y., Hao Y., Cai Y., Cheng Z., Deng C., Liu X. (2019). Neuroprotective Effect of Glycosides in Buyang Huanwu Decoction on Pyroptosis Following Cerebral Ischemia-Reperfusion Injury in Rats. J. Ethnopharmacol..

[42] Hu J.J., Liu X., Xia S., Zhang Z., Zhang Y., Zhao J., Ruan J., Luo X., Lou X., Bai Y. (2020). FDA-Approved Disulfiram Inhibits Pyroptosis by Blocking Gasdermin D Pore Formation. Nat. Immunol..

[43] Wang P.-C., Wang S.-X., Yan X.-L., He Y.-Y., Wang M.-C., Zheng H.-Z., Shi X.-G., Tan Y.-H., Wang L.-S. (2022). Combination of Paeoniflorin and Calycosin-7-Glucoside Alleviates Ischaemic Stroke Injury via the PI3K/AKT Signalling Pathway. Pharm. Biol..

[44] Xiao L., Dai Z., Tang W., Liu C., Tang B. (2021). Astragaloside IV Alleviates Cerebral Ischemia-Reperfusion Injury through NLRP3 Inflammasome-Mediated Pyroptosis Inhibition via Activating Nrf2. Oxid. Med. Cell. Longev..

[45] Zhang S., Jin X., Zhang Y., Zhou X., Gao W. (2022). Protective Effects of Different Doses of Calycosin from Astragalus on Neuronal Cells of Rats with Cerebral Ischemia-Reperfusion Injury. J. Hebei Tradit. Chin. Med..

[46] Yuan H., Han Q., Yu H., Yu Y., Liu X., Xue Y., Li Y. (2025). Calycosin Treats Acute Myocardial Infarction via NLRP3 Inflammasome: Bioinformatics, Network Pharmacology and Experimental Validation. Eur. J. Pharmacol..

[47] Liu W., Zhang Y., Chen J., Zeng K., Zhou X. (2022). Simultaneous Content Determination of 10 Components in Lyophilized Product of Buyang Huanwu Decoction on UPLC. Tradit. Chin. Drug Res. Clin. Pharmacol..

[48] Longa E.Z., Weinstein P.R., Carlson S., Cummins R. (1989). Reversible Middle Cerebral Artery Occlusion without Craniectomy in Rats. Stroke.

