# Supplementary material for: Study on the Mechanism of Buyang Huanwu Decoction in Treating Ischemic Stroke by Regulating the NLRP3/Caspase-1 Signaling Pathway
Source: Pharmaceuticals (Basel). 2026 Apr 1;19(4):567. doi: 10.3390/ph19040567 (PMC13118797; doi:10.3390/ph19040567)
Supplement: Supplementary file 1 [file pharmaceuticals-19-00567-s001.zip › pharmaceuticals-4160334-supplementary.pdf]

Supporting Information

Study on the Mechanism of Buyang Huanwu Decoction in Treating Ischemic Stroke by Regulating the NLRP3/Caspase-1 Signaling Pathway

Keqi Zeng †, Cong Nie †, Xin Zhou, Die Pei, Jieyi Huang and Yingfeng Zhang *

School of Chinese Materia Medica, Guangzhou University of Chinese Medicine, University Town,
Panyu District, Guangzhou 51006, China
* Correspondence: zhangyingfeng@gzucm.edu.cn
† These authors contributed equally to this work.

Contents

Figure S1. HPLC fingerprint of BHD

Figure S2. Systematic cluster analysis of 16 batches of BHD

Table S1. The fingerprint gradient elution procedur of BHD lyophilized product

Table S2. The gradient elution procedur of rabbit's BHD-CCSF detection

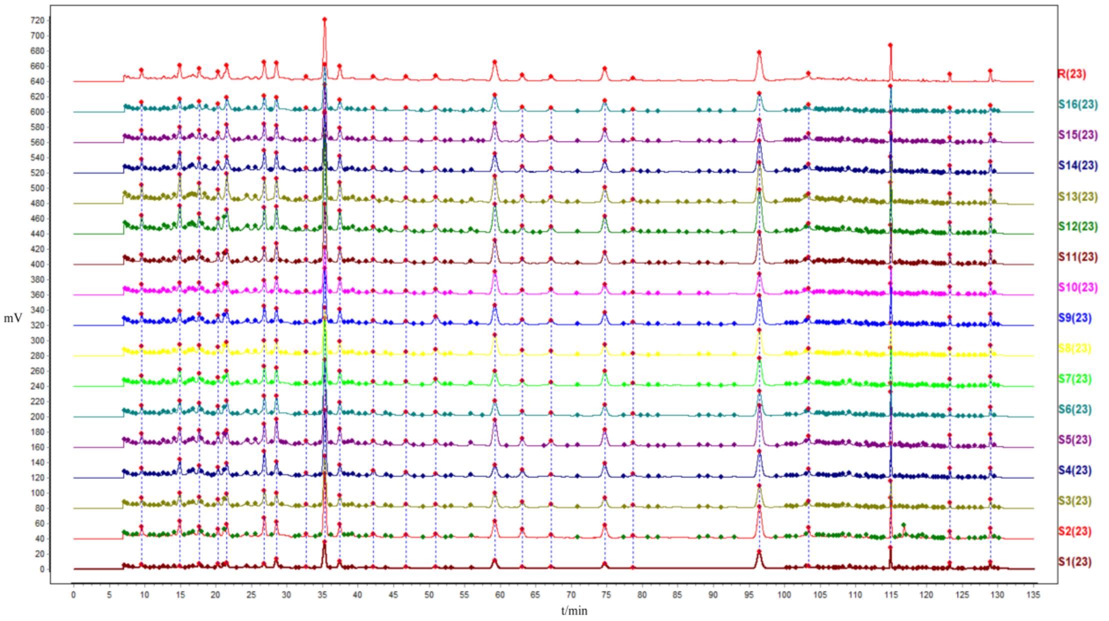

Figure S1. HPLC fingerprint of BHD

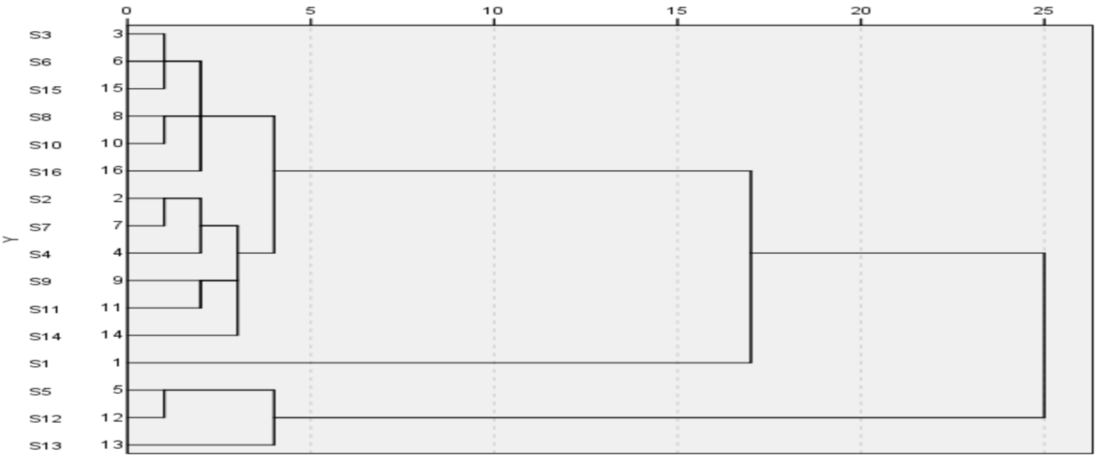

Figure S2. Systematic cluster analysis of 16 batches of BHD

Table S1. The fingerprint gradient elution procedur of BHD lyophilized product

Time/min	A/%	B%
0	90	10
3	90	10
7	87	13
32	82.6	17.4
42	82.5	17.5
50	82	18
90	77	23
108	55	45
118	40	60
125	40	60

130	90	10
135	90	10

Table S2. The gradient elution procedur of rabbit's BHD-CCSF detection

Time/min	A/%	B%
0	90	10
5	65	35
10	35	65
13	15	85
15	0	100
17	0	100
18	90	10
21	90	10
